# How Different Molecular Markers Estimate the Diversity of European Species of the *Ganoderma* Genus

**DOI:** 10.3390/jof9101023

**Published:** 2023-10-17

**Authors:** Peter Pristas, Terezia Beck, Lea Nosalova, Svetlana Gaperova, Jan Gaper

**Affiliations:** 1 Institute of Biology and Ecology, Pavol Jozef Safarik University in Kosice, Srobarova 2, 04154 Kosice, Slovakia; lea.nosalova@upjs.sk; 2 Centre of Biosciences, Institute of Animal Physiology, Slovak Academy of Sciences, Soltesovej 4–6, 04001 Kosice, Slovakia; 3 Department of Biology and Ecology, Faculty of Natural Sciences, Matej Bel University, Tajovskeho 40, 97401 Banska Bystrica, Slovakia; gasparcova.terezka@umb.sk (T.B.); gaperova.svetlana@umb.sk (S.G.); 4 Department of Biology and General Ecology, Faculty of Ecology and Environmental Sciences, Technical University, T. G. Masaryka 24, 96053 Zvolen, Slovakia; jan.gaper@tuzvo.sk

**Keywords:** fungal diversity, molecular markers, polypores, *Ganoderma*, Europe

## Abstract

Based on published anatomical-morphological and ecological characteristics and phylogenetic evidence, six species of the *Ganoderma* genus are known to occur in Europe, namely, *G. applanatum* (Pers.) Pat., *G. adspersum* (Schulzer) Donk, *G. pfeifferi* Bres., *G. resinaceum* Boud., *G. carnosum* Pat., and *G. lucidum* (Curtis) P. Karst. Molecular markers (DNA sequences of selected genes or intergenic spacers) revolutionized our view of fungal variability. Every one of the four most frequently used molecular markers (ITS (internal transcribed spacer) and partial sequences of LSU (rRNA large subunit), tef1-α (translation elongation factor 1-alpha), and Rpb2 (RNA polymerase II second largest subunit)) provides a different view on the variability of European species of the *Ganoderma* genus. Both the lowest intraspecies variability and the best species differentiation (interspecies divergence) were observed for the LSU marker, and based on our data, this marker could be recommended for identification and species delineation in European *Ganoderma* spp. The sequences of the most frequently used ITS marker were unable to discriminate between *G. lucidum* and *G. carnosum,* and in general, this species pair showed the lowest interspecies divergence using all markers tested. Surprisingly, up to now, hidden variability has been detected in several *Ganoderma* spp., indicating the existence of possible cryptic taxa within the European *Ganoderma* morphospecies.

## 1. Introduction

*Ganoderma* P. Karst. (Polyporaceae, Basidiomycota) is a large and cosmopolitan genus of fungi that cause white rots of dead and living hardwood and conifer trees and shrubs in both urban and natural surroundings. The genus includes several phytopathologically important species whose economic impact is substantial. Despite this pathogenic nature, many species, especially taxa formerly identified as *Ganoderma lucidum* s.l. in Asia, are believed to possess pharmaceutical properties and have been used in traditional Asian medicine for millennia [1].

The genus *Ganoderma*, created by P. Karsten in 1881 with *Polyporus lucidus* Curtis: Fr. [2] and typified by *Ganoderma lucidum* (Curtis) P. Karst., is currently characterized by sessile to stipitate basidiomata with a thick, dull crust or shiny, thin cuticle on the pileus surface, a trimitic hyphal structure generally with clamped generative hyphae, and narrowly to broadly ellipsoid double-walled basidiospores with interwall pillars, which are non-dextrinoid [3,4,5,6,7].

Enormous variability in the macroscopic characters of basidiomata and their relatively uniform microscopic characters in many *Ganoderma* species have resulted in large confusion in the taxonomy of this genus for the past 30 years. As a result, many synonyms were created, or vice versa, the wide species concept was used, and different species were merged. Both the difficulties in determination and the different species concepts have resulted in ambiguous species delimitation and identification systems [5]. Currently, there are 492 records of taxa in the Index Fungorum (http://www.indexfungorum.org/, accessed on 16 September 2023) and 535 records of taxa (482 with legitimate status) in MycoBank (http://www.mycobank.org/, accessed on 16 September 2023). In the early 1990s, before the era of molecular analysis, a total of 148 *Ganoderma* species were accepted [2], of which some probably represent different morphospecies only [8]. However, over the past three decades, with the progress made through molecular techniques [9,10,11,12,13], the species complexes are being elucidated, although this advancement is slow [13]. Currently, 180 *Ganoderma* species are accepted, whereas nearly 500 species are estimated globally, of which 60% are awaiting discovery [14].

Across climatic regions, the greatest variability in the *Ganoderma* genus is seen in tropical and subtropical regions of Africa, America, Asia, and Oceania [3], while the variability in Europe seems to be the lowest [3,4,5,6,7]. Although more than 20 *Ganoderma* species have been described from Europe [2], only seven morphospecies, based on classical methods of fungal identification, which rely mainly on comprehensive micro- and macromorphological characteristics, ecological features, and distributional patterns, are accepted, namely, *Ganoderma applanatum* (Pers.) Pat., *Ganoderma adspersum* (Schulzer) Donk, *Ganoderma pfeifferi* Bres., *Ganoderma resinaceum* Boud., *Ganoderma carnosum* Pat., *Ganoderma valesiacum* Boud., and Ganoderma *lucidum* (Curtis) P. Karst. [3,4,5,6,7,15]. In the scientific literature, *G*. *applanatum* appears under the name *Ganoderma lipsiense* sensu auct., *G*. *adspersum* under the name *Ganoderma australe* (Fr.) Pat. sensu Ryvarden et Melo p. p. (in Europe), and *G*. *pfeifferi* under the name *Ganoderma cupreolaccatum* Z. Igmándy, and, finally, *G*. *carnosum* appears under the name *Ganoderma atkinsonii* H. Jahn, Kotl. and Pouzar. Phylogenetic analyses based on internal transcribed spacer (ITS) sequences later confirmed that *Ganoderma valesiacum* is not a valid species within this genus, and in this study, the taxon is considered a taxonomic synonym of *G. carnosum.*

Based on phylogenetic evidence, a total of five well-separated clades are currently known from Europe, namely, *G*. *applanatum*, *G*. *adspersum*, *G*. *pfeifferi*, *G*. *lucidum,* and *G*. *carnosum,* in accordance with the morphospecies concept. Further studies are needed to clarify the species boundaries within the sixth European *G*. *resinaceum* clade [12].

Among European species, *G*. *pfeifferi* is easily recognized in the field by the unique features of the perennial basidioma with a resinous layer on the pileus and dark brown context [15]. Recent phylogenetic studies [12,13], consistent with morphological examinations, demonstrated that the names *G*. *cupreolaccatum* and *G*. *pfeifferi* are conspecific [15].

*G*. *applanatum* can also be easily recognized by the characteristic continuous tube strates with intervening layers of context when the basidiomata are more than two years old [16]. *G*. *adspersum* has similar perennial basidiomata without intervening layers of context, and the basidiospores are larger in this species. If the collected basidiomata are too young, molecular tools are always required to be sure of their exact identification [16]. *Ganoderma resinaceum*, when growing on woody plants in a very humid habitat, sometimes has stipitate basidiomata and presents a thinner context when compared to the specimens usually growing in other habitats [12,17]. In this case, the basidiomata of *G*. *resinaceum* may be confused with those of *G*. *lucidum*, but these two species diverge from one another, both in what their spores look like [2,16] and genetically [12]. The *G*. *lucidum* basidiospores appear more coarsely warted than those of *G*. *resinaceum*. In addition to these, as has been mentioned above, further studies are needed to clarify the species boundaries of two probably cryptic species within the sixth *G*. *resinaceum* clade [12,14,18].

Three of the seven European morphospecies (*G*. *lucidum* s.str., *G*. *carnosum*, and *G*. *valesiacum*) have been members of the *G*. *lucidum* complex over the last decades [5,19]. *G*. *carnosum* has a central to southern European distribution [7], and the species has been recorded from Great Britain, all of central Europe to the Carpathian Mts. in Ukraine, and from the Mediterranean area [3]. The species is most associated with conifers, rarely hardwoods. *G*. *lucidum* is a common morphospecies, most associated with hardwoods, rarely conifers, and recorded in most European countries [3,7]. According to Bernicchia and Gorjón [7], the best way to distinguish these two morphospecies is by applying a KOH solution to a thin resin layer on the cutis of their basidiomata. In *G*. *lucidum,* this layer dissolves; in *G*. *carnosum,* it does not [7]. A rare species, *G*. *valesiacum*, more common only in some valleys of the Alps and Dolomites [7], is known to occur from Great Britain over central Europe to eastern Russia and from three Mediterranean countries, namely Portugal, France, and Italy [3,7,20]. *G*. *valesiacum* is the only *Ganoderma* species, with larch as the only host. Firstly, based on classical methods of fungal determination, such as comparative-morphological studies of type specimens of the taxa in question, collections from nature localities, and the analysis of literature data, *G*. *valesiacum* and *G*. *carnosum* were treated as *G*. *lucidum* varieties [19]. Therefore, this study places *G*. *valesiacum* and *G*. *carnosum* as taxonomic synonyms of *G*. *lucidum* [3]. Similarly, preliminary molecular approaches indicated that *G*. *carnosum* and *G*. *valesiacum* cannot be separated from *G*. *lucidum* on the species level [5]. In addition, according to data compiled by Ryvarden and Mello [3], the taxonomic placement of *G*. *valesiacum* is uncertain because its microstructure is similar to that of *G*. *lucidum,* and the only criterion for dividing them seems to be the host preferences only for *Larix*. Finally, sequencing techniques are essential to see whether this is only an infraspecific taxon of *G*. *lucidum* and to establish its relationship to *G*. *carnosum*, differentiated primarily because of its preference for conifers (*Abies alba* Mill. and *Picea abies* (L.) H. Karst.) [3]. Recently, judging by ITS sequences from Slovakia [12], Italy [16], and the Czech Republic [21], European *G*. *carnosum* was shown to be identical to *G*. *valesiacum*, although they produce quite different mycelia in pure cultures [16]. Furthermore, an ITS-based phylogeny indicated that *G*. *carnosum* is identical with *G*. *oregonense* Murrill, a species with a North American origin [7,22]. Moreover, *G*. *tsugae* Murrill, also a North American species, and *G*. *lucidum* are only slightly different in sequence and may be conspecific in topology [21].

Advances in DNA technologies allowed the application of modern molecular methods for the identification of fungi. Molecular DNA markers (DNA sequences of selected genes or intergenic spacers) provide a better alternative method than traditional morphological methods. For the rapid identification of fungi, ITS (internal transcribed spacer) located between 18S and 28S nrDNA [23] is the most frequently used molecular marker as it provides acceptable resolution in numerous taxa. Nonetheless, to overcome the insufficient resolution of ITS sequences in several closely related taxa, several other marker genes were proposed for fungi identification. These include some protein-encoding genes, e.g., translational elongation factor 1α (tef-1α), DNA-directed RNA polymerase II largest (Rpb1) and second largest (Rpb2) subunits, β-tubulin II (tub2), DNA topoisomerase I (top1) or ribosomal RNA genes, and intergenic spacers, e.g., 28S nrDNA (LSU), 18S nrDNA (SSU), and intergenic spacer (IGS) [24]. Each of these markers provides a different level of fungal identification reliability. For the fine-scale identification of fungi, concatenated alignment of the ITS region with one or more protein-coding genes may be effective [25], but this approach will probably be rapidly replaced by phylogenomics [26] and whole genome comparisons due to the rapid progress in sequencing technologies.

This paper discusses the current taxonomical state of European species of the *Ganoderma* genus and provides a new view on the diversity of European species of the *Ganoderma* genus in the light of different molecular markers used.

## 2. Materials and Methods

All valuable information on observed morpho-anatomical characteristics, geographical distribution, habitat preference, and classification of European species of the *Ganoderma* genus was gathered through evaluation of literature and searches in online databases using SciFinder and Web of Knowledge, primarily from well-established monographs [3,4,5,6,7] and original species descriptions.

In the nomenclature of fungi, the Index Fungorum [27] database was followed. In the nomenclature of woody plants, the International Plant Names Index database [28] was followed.

From multiple *Ganoderma* spp. sequence data in the GenBank database (more than 65,000 entries of *G. adspersum, G. applanatum, G. carnosum, G. lucidum, G. pfeifferi,* and *G. resinaceum*), a custom dataset was generated consisting exclusively of *Ganoderma* spp. sequences of European origin (European countries, including Turkey, in the “country” field). Uncultured and environmental sample sequences were removed from the analysis, as were entries with more than 3 ambiguous bases in the sequence. For subsequent analyses, 4 DNA markers were selected: ITS, LSU, tef1-α, and Rpb2, with the highest number of entries (Table 1). For ITS markers, sequences containing both ITS1 and ITS2 regions were used only. The final dataset comprised 312 ITS sequences, 62 LSU sequences, 64 tef1-α sequences, and 71 Rpb2 sequences (Table 1). In the dataset, the most represented were ITS sequences, as this marker is a “gold standard” used for fungi identification. For every marker intraspecific (Table 1) and for all species pairs interspecific (Table 2), divergence values were calculated.

The sequences were aligned using the MUSCLE algorithm implemented in MEGA11 software [29]. Intra- and interspecies distances were calculated using the Kimura 2-parameter model [30]. All ambiguous positions were removed for each sequence pair. The evolutionary relatedness of sequences was inferred using the neighbor-joining method [31], and the evolutionary distances were computed using the Kimura 2-parameter method [30], and all ambiguous positions were removed for each sequence pair. Bootstrap confidence values were obtained by applying 500 replications. All sequence analyses were conducted in MEGA11 [29].

## 3. Results and Discussion

To estimate the diversity of European species in the *Ganoderma* genus, sequences of different molecular markers were downloaded from the GenBank database and compared. Due to the lack of sequences of several loci from the same specimen or isolate, no multi-locus phylogenetic comparisons were performed. The single-gene comparisons showed different levels of interspecies divergence in *Ganoderma* spp. (Table 2).

The “good” molecular marker for species Identification and delineation should have the lowest possible intraspecies variability and the highest possible interspecies divergences. In several cases, practically no divergence was observed, e.g., ITS marker for *G. applanatum* species or tef-1α marker for *G. adspersum* and *G. carnosum*. From the markers analyzed in this work, the lowest intraspecies divergence was observed for the LSU marker, with an average level of variability of less than one nucleotide substitution per thousand nucleotides (Table 1). Based on our data, this marker could be recommended for identification and species delineation in European *Ganoderma* spp. The average interspecies divergences observed for this marker were eleven times higher (11.2 nucleotide substitutions per 1000 nucleotides) compared to intraspecies divergences. All other markers showed at least five times higher average levels of intraspecies divergence. The ITS marker showed a higher level of intraspecies diversity in all but *G. applanatum* species, but the highest level of interspecies divergence was observed for the Rpb2 marker. Another protein-encoding gene (partial sequences of the tef-1α gene) showed a two-fold lower level of intraspecies diversity compared to the ITS marker. This marker provided the best ratio of inter/intraspecies diversity (about 20), but it also showed the existence of two types of sequences in *G. lucidum* species.

**Figure 1:**
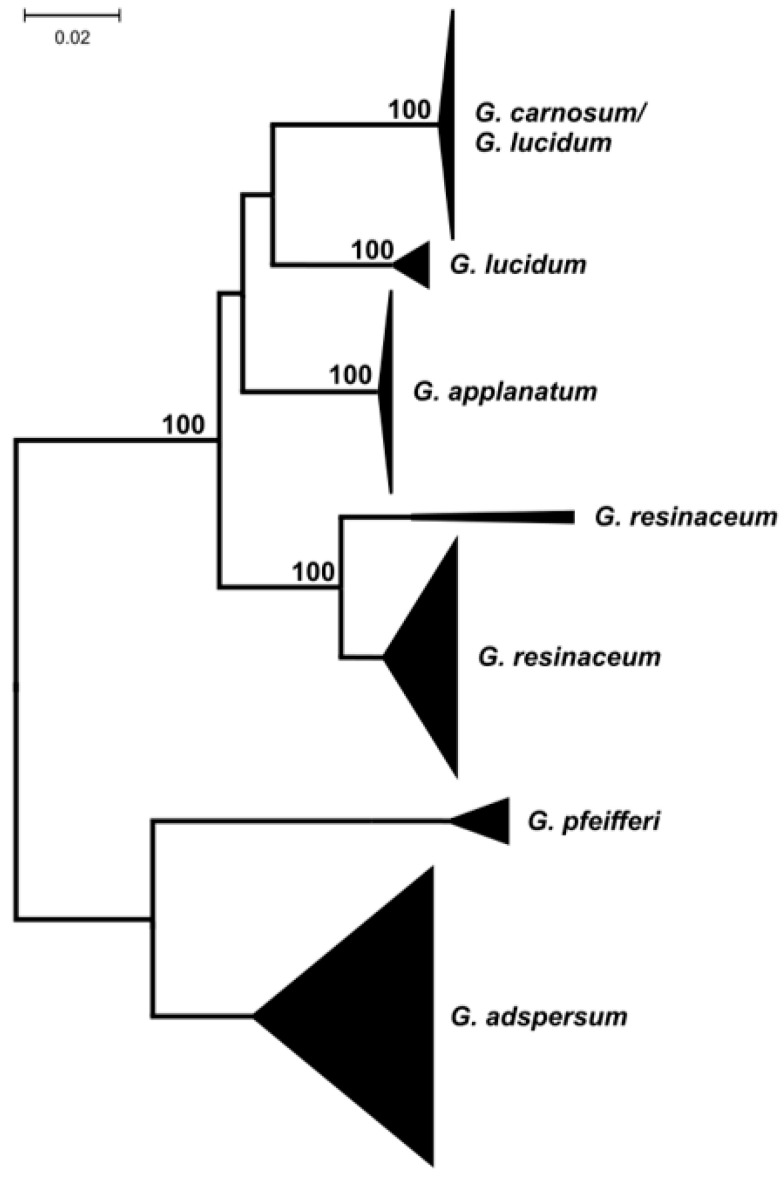
Dendrogram showing relatedness of ITS sequences of European species of the *Ganoderma* genus. The length of the triangle corresponds to the intraspecies divergence within species, and the height of the triangle corresponds to the number of sequences available. Numbers at nodes are bootstrap values after 500 replications (only bootstrap values over 75 are shown). The scale is in the number of nucleotide substitutions per 1000 nucleotides.

The highest interspecies divergences were observed, as expected, for the non-coding ITS marker, followed by tef-1α, Rpb2, and the LSU marker. The lowest interspecies differences were observed for the *G. lucidum*/*G. carnosum* species pair belonging to the *G. lucidum* complex [5,19] using all markers analyzed. For the ITS sequence marker, multiple sequence comparisons (Figure 1) showed that this pair of species cannot be differentiated using this marker.

**Figure 2:**
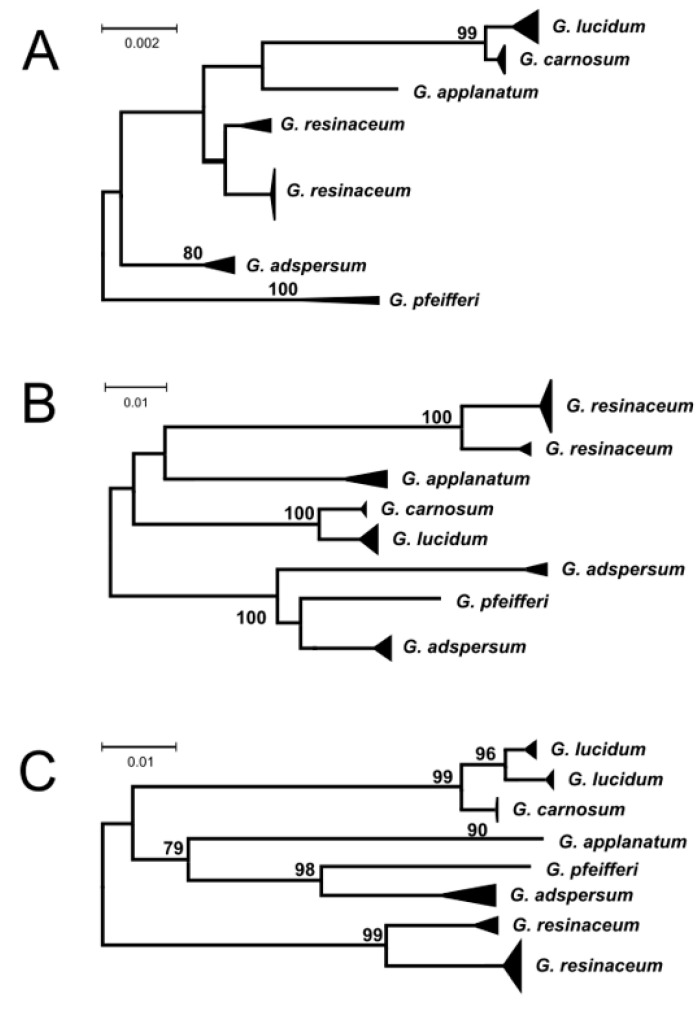
Dendrogram showing relatedness of LSU (**A**), Rpb2 (**B**), and tef1-α (**C**) sequences of European species of the *Ganoderma* genus. The length of the triangle corresponds to the intraspecies divergence within species, and the height of the triangle corresponds to the number of sequences available. Numbers at nodes are bootstrap values after 500 replications (only bootstrap values over 75 are shown). The scale is in the number of nucleotide substitutions per 1000 nucleotides.

All other markers used were able to safely resolve the *G. lucidum* and *G. carnosum* species pairs (Figure 2).

The sequence comparison of the ITS sequences of *G. lucidum* and *G. carnosum* led to the identification of two well-separated types of *G. lucidum* ITS sequences. While one group consists exclusively of *G. lucidum* sequences, the second one is composed of both *G. lucidum* and *G. carnosum* sequences, sharing practically identical sequences. Taking into account the relatively high number of sequences in this mixed group (18 *G. carnosum* and 56 *G. lucidum* sequences), it is rather improbable that the observed grouping is the result of the misidentification of specimens, and it is probably evidence of genetic non-homogeneity within European *G. lucidum*. However, morphological differentiation of the species in the *G. lucidum* complex has some limitations, and the application of potassium hydroxide solution onto a thin resin layer on the cutis of their basidiomata, as mentioned earlier, is the best way to distinguish between these two species [16]. The existence of two (geno)types among *G. lucidum* sequences was supported by the tef1-α sequence comparison (Figure 2C), but for no other molecular markers, *G. carnosum* and *G. lucidum* sequences fell into the same branch.

Similar genetic non-homogenity (the placement of sequences of one species in two different branches) was observed for several other analyzed species. All molecular markers supported the existence of two genotypes in *G. resinaceum* species, as already reported by Naplavova et al. [18]. The Rpb2 marker provided the most complex view of variability in European *Ganoderma* spp. The existence of two different genotypes within the species was observed not only for *G. resinaceum* but in *G. adspersum* as well and could be evidence of cryptic speciation in *Ganoderma* spp. However, no other molecular marker supported the existence of two genotypes, and there is no other non-homogeneity indication of the Southern bracket (*G. adspersum*) in scientific literature. Theoretically, the heterogeneity within these morphospecies could be due to very wide and quite different host spectra in different regions [32], and so far, the only collection from France showed morphological features of the species [6]. The existence of such cryptic species, which cannot be easily distinguished morphologically but which possess significantly diverged sequences of molecular markers, has been proposed in several fungal genera [33,34,35,36].

Among six currently accepted *Ganoderma* morphospecies known from Europe, molecular methods recognized seven, seven, eight, and eight genotypes using ITS, LSU, Rpb2, and tef1-α markers, respectively, indicating that morphological approaches probably underestimate the diversity of European species of the *Ganoderma* genus.

The analysis of available *Ganoderma* spp. sequences of European origin showed that the sequencing of a sufficiently long (about 1000 bp) fragment of the 28S ribosomal RNA gene (large subunit ribosomal RNA) provides the best way for the identification of European species of the *Ganoderma* genus. Widely used ITS sequences are unable to resolve between (some) *G. lucidum* and *G. carnosum* species, and all other molecular markers used see additional, up to now hidden variability, indicating probably the cryptic speciation within the European *Ganoderma* morphospecies. Other analyses involving multi-locus or whole genome comparisons will be necessary to reveal the true diversity of European species of *Ganoderma* spp.

## Figures and Tables

**Table 1:** Intraspecies divergence of different molecular marker sequences in *Ganoderma* spp. originated in Europe. The variability is shown as the number of base substitutions per site from averaging over all sequence pairs within each group. (na—not applicable due to the low number of sequences available).

Species	Marker							
	ITS		LSU		tef1-α		Rpb2	
	No. Sequences	Diversity	No. Sequences	Diversity	No. Sequences	Diversity	No. Sequences	Diversity
*G. adspersum*	85	0.012	7	0.000	9	0.000	16	0.033
*G. applanatum*	57	0.000	1	na	1	na	7	0.009
*G. carnosum*	19	0.006	12	0.000	11	0.000	5	0.001
*G. lucidum*	65	0.025	13	0.000	13	0.006	12	0.003
*G. pfeifferi*	13	0.002	3	0.002	1	na	1	na
*G. resinaceum*	70	0.008	26	0.000	29	0.013	27	0.010
average		0.009		0.000		0.005		0.011

**Table 2:** The interspecies divergence of different molecular marker sequences in *Ganoderma* spp. originated in Europe. The divergence is shown as the number of base substitutions per site from averaging over all sequence pairs between groups. The divergence values are shown in order: ITS, LSU, tef1-α, and Rpb2. The nested table shows the average interspecies divergence for every molecular marker tested.

Species	Marker	*G. adspersum*	*G. applanatum*	*G. carnosum*	*G. lucidum*	*G. pfeifferi*	*G. resinaceum*
*G. adspersum*	ITS	-					
	LSU	-					
	tef1-α	-					
	Rpb2	-				marker	average
*G. applanatum*	ITS	0.194	-			ITS	0.154
	LSU	0.009	-			LSU	0.010
	tef1-α	0.093	-			tef1-α	0.099
	Rpb2	0.092	-			Rpb2	0.067
*G. carnosum*	ITS	0.201	0.083	-			
	LSU	0.014	0.010	-			
	tef1-α	0.106	0.108	-			
	Rpb2	0.102	0.091	-			
*G. lucidum*	ITS	0.199	0.076	0.022	-		
	LSU	0.015	0.011	0.002	-		
	tef1-α	0.112	0.109	0.016	-		
	Rpb2	0.106	0.086	0.016	-		
*G. pfeifferi*	ITS	0.181	0.194	0.204	0.204	-	
	LSU	0.011	0.018	0.016	0.017	-	
	tef1-α	0.054	0.091	0.108	0.110	-	
	Rpb2	0.055	0.088	0.096	0.104	-	
*G. resinaceum*	ITS	0.224	0.099	0.110	0.105	0.217	-
	LSU	0.006	0.007	0.010	0.010	0.012	-
	tef1-α	0.115	0.114	0.110	0.119	0.119	-
	Rpb2	0.128	0.107	0.106	0.109	0.126	-

## Data Availability

In the manuscript, the sequence data publicly available from the NCBI GenBank database were used.
